# Early changes in corticospinal excitability for subliminally presented fearful body postures

**DOI:** 10.1038/s41598-025-13185-y

**Published:** 2025-08-08

**Authors:** Sara Borgomaneri, Thomas Quettier, Marianna Ambrosecchia, Simone Battaglia, Marco Tamietto, Alessio Avenanti

**Affiliations:** 1 Centro studi e ricerche in Neuroscienze Cognitive, Dipartimento di Psicologia “Renzo Canestrari”,, Alma Mater Studiorum Università di Bologna, Campus di Cesena, Cesena, 47521 Italy; 2 Department of Psychology, University of Torino, Turin, Italy; 3 Centro de Investigación en Neuropsicología y Neurociencias Cognitivas, Universidad Católica del Maule, Talca, Chile; 4 Department of Psychology “Renzo Canestrari”, University of Bologna, Viale Berti Pichat 5, Bologna, 40127 Italy

**Keywords:** Emotion, Neurophysiology

## Abstract

Fearful body expressions convey critical information that is rapidly and preferentially processed, facilitating swift motor reactions to potential dangers. Consistent evidence has shown that even the subliminal presentation of fear-related expressions can impact visual processing and autonomic responses, increasing sensory vigilance for monitoring potential threats. However, it remains unclear whether the presentation of non-visible emotional bodies modulates corticospinal excitability (CSE) in the observer. To investigate this, we asked 22 healthy participants to perform a sex discrimination task involving neutral target body postures, preceded by the brief subliminal presentation of fearful, happy, or neutral body postures. CSE was tested using Transcranial Magnetic Stimulation (TMS) at early time points (70, 90, and 110 ms) after target stimulus onset. Results showed a significant CSE reduction in the dominant hemisphere for subliminal fearful primes compared to happy and neutral primes. This CSE suppression was independent of the time of stimulation, participants’ subjective or objective awareness, metacognitive sensitivity, or personality traits. Our findings highlight an early automatic activation of the motor system in response to subliminal fearful stimuli, supporting the view that fearful expressions, even when not consciously perceived, activate basic survival mechanisms for monitoring and preparing fast motor responses to potential threats.

**Supplementary Information:**

The online version contains supplementary material available at 10.1038/s41598-025-13185-y.

## Introduction

Consistent evidence has shown that negative stimuli signaling potential danger can be processed outside conscious awareness, thereby facilitating rapid threat detection and bolstering protective behaviors^1–3^. Among these, fearful expressions stand out as particularly salient social cues, conveying the presence of a possible threat in the surroundings, without specifying its source^3,4^. Because of this ambiguity, fearful expressions are thought to serve as a general alarm signal, prompting heightened vigilance and orienting attention toward potential danger in the surroundings. Interestingly, unattended or unseen fearful expressions from faces or bodies, either due to experimental manipulation or permanent brain damage, modulate spontaneous expressive reactions, physiological changes, or subsequent judgments to consciously reported stimuli^5–14^. For example, Zhan and de Gelder^15^ demonstrated that subliminal perception of fearful body postures, using continuous flash suppression, enhanced conscious discrimination of visible angry expressions. This finding supports the notion that fearful stimuli capture attention and facilitate processing other negative emotions, indicating a complex neural prioritization for unconscious fear perception in rapid threat detection.

Collectively, neuroimaging studies in both healthy and brain-damaged patients point to a subcortical network for non-conscious thereat detection centered on the amygdala. Concerning the timing, magnetoencephalography (MEG) studies have identified a dissociation in amygdala responses to threat signals. Early amygdala responses (~ 60 ms post-stimulus onset) occur automatically, independent of conscious attention, while later responses (~ 280 ms) are modulated by voluntary attention^16^. More conclusively, human intracranial electrophysiology found rapid responses (~ 70 ms) in the lateral amygdala, specifically to fearful facial expressions, which occur much earlier than similar fear responses in the visual cortex, typically emerging in the temporal regions around 170 ms post-stimulus^17^.

Beyond increasing sensory vigilance at pre-attentive or non-conscious processing stages, fearful expressions consistently influence the motor system by engaging preparatory responses such as freezing or orienting immobility^18–23^. These states, which involve a transient suppression of overt movements, are widely recognized as active defensive strategies that enhance perceptual sensitivity and facilitate rapid transitions to fight-or-flight responses when necessary^24–27^.

Our previous studies have demonstrated that conscious perception of negative scenes^28^, facial expressions^20,29^, or emotional bodily postures rapidly affects motor excitability^18–23,30^. In particular, observing fearful body expressions has been found to consistently suppress motor excitability^18,23,31^. On the other hand, Quettier et al.^32^ used a modified Stop Signal Task with subliminal emotional primes and showed that undetected fear stimuli enhanced participants’ ability to inhibit motor responses, implying an indirect modulation of motor excitability. However, only one study directly investigated whether subliminally presented emotional stimuli can modulate corticospinal excitability (CSE), a reliable measure of the motor system’s functional state that reflects shifts in the balance between excitatory and inhibitory activity in response to experimental manipulations^33^. In two different experiments, the authors presented fearful, angry, neutral bodies or objects, with TMS pulses delivered over the left primary motor cortex (M1) at different time points post-stimulus onset (200, 400, 500, 700 ms), but found no conclusive evidence of non-conscious fearful stimuli altering CSE. Therefore, it remains unclear whether subliminally presented fearful stimuli can modulate CSE, particularly at early time windows, as shown for consciously perceived stimuli.

To address these gaps, we investigated whether CSE could be implicitly modulated by rapid (16 ms) masked presentations of emotional bodily expressions while participants performed a sex categorization task on neutral body postures. We probed CSE by stimulating both the left and right M1 at 70, 90, and 110 ms post-stimulus – time points corresponding to the latency of the P1, the earliest cortical ERP component that is consistently modulated by fearful expressions^34–36^. Previous studies by our group have demonstrated a reduction in CSE within the 70–90 ms^23^, 100–125 ms^21^ and 150 ms^22^ intervals, as well as decreased intracortical facilitation (ICF) at 100–125 ms^18^, thus indicating that consciously perceived fearful stimuli reduce motor excitability in this early post-stimulus time window^18,21,23^. In line with the previous findings, we considered the selected time points (70, 90, 110 ms) as the ideal latencies to investigate whether CSE can be also affected by subliminally presented fearful stimuli. Finally, building on our previous TMS evidence^18,21,23^, no time-specific effects are expected, as earlier studies showed inhibitory activity across overlapping time intervals rather than at distinct latencies, suggesting a temporally broad modulation of motor excitability within the early post-stimulus window (70–110 ms).

Given the evolutionary need to respond quickly to impending signals of fear, often manifesting in freezing, we hypothesized that subliminal fearful bodily postures would reduce CSE relative to happy and neutral postures, even when task-unrelated. We also assessed prime visibility in a separate session (awareness check) to test whether individual differences in stimulus awareness might impact CSE. Consistent with our predictions, fearful bodily expressions reduced CSE in the left dominant hemisphere across all tested TMS time points, regardless of participants’ awareness of the prime stimuli. These results suggest that fearful bodily expressions are a privileged signal influencing motor readiness both rapidly and independently of conscious perception.

## Methods

### Participants

Twenty-two healthy young adults were involved in the study (11 female, mean age ± standard deviation: 24.45 years ± 2.42). The sample size was established using G*Power software^37^ by conducting a power analysis that aimed for a high power (1 e b) of 0.95, while keeping the significance threshold (a) at 0.05. Based on the MEP modulations patterns observed in our prior work^18,19,23,38^, we expected a MEP decrease in response to Prime Body Expressions during the task, with a large effect size (f = 0.4). This sample size was also consistent with the typical sample used across ten experimental groups in the aforementioned studies, each typically consisting of 15 participants. The experiments were carried out at the Center for studies and research in Cognitive Neuroscience, Department of Psychology, University of Bologna. All participants were right-handed, unaware of the study’s objectives, and reported no history of neurological or psychiatric conditions, visual impairments, medicine intake, or contraindications for TMS^39^. The University of Bologna’s Bioethics Committee approved the study, which was conducted in compliance with legal requirements and international guidelines (Declaration of Helsinki, 2013). Prior to the experiment, all participants gave their informed consent and received a brief overview of the experimental procedures. No discomfort or adverse effects related to TMS were reported or observed.

### Visual stimuli

**Fig. 1:**
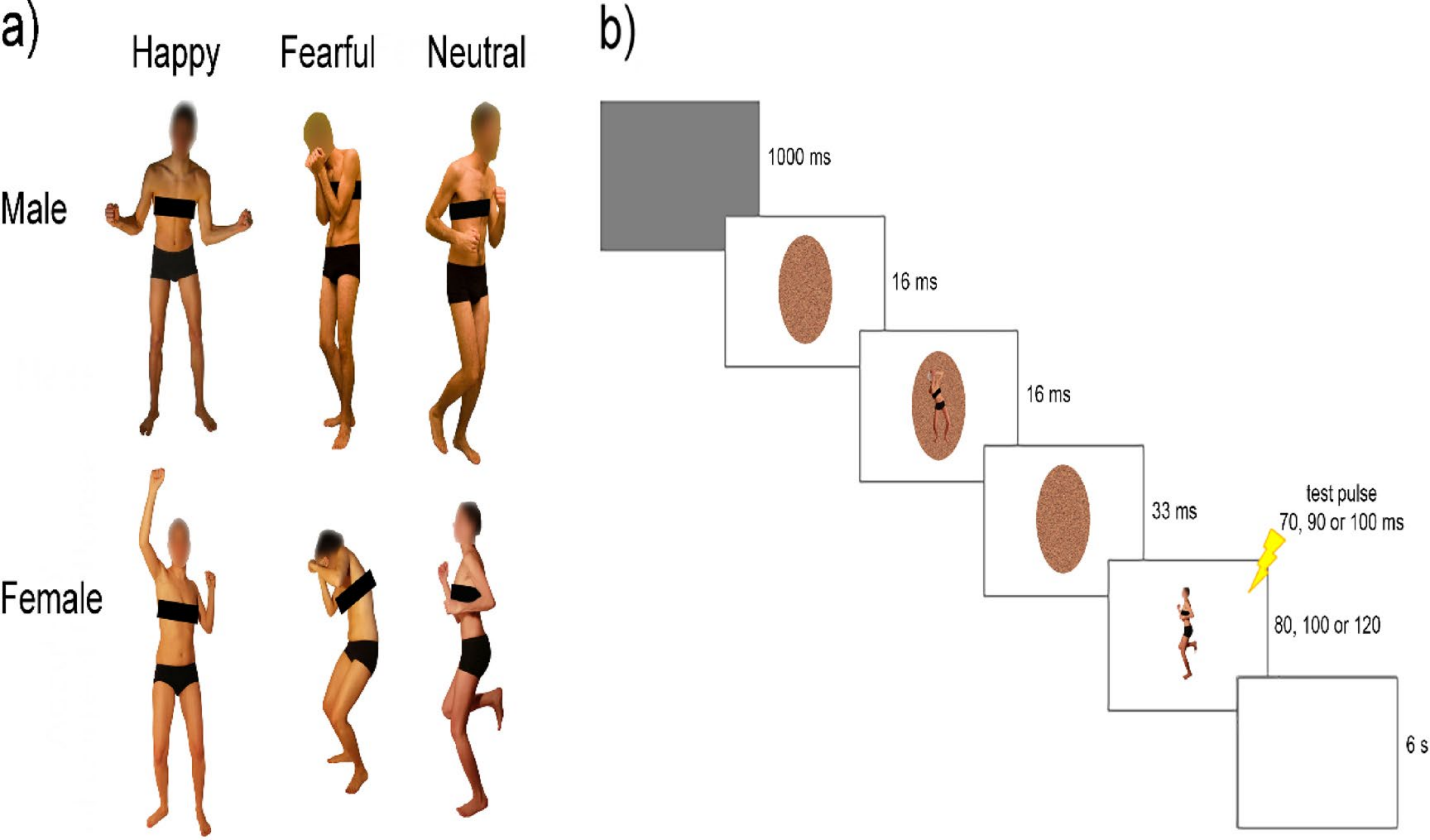
(**A**) Examples of male and female body stimuli showing happy, fearful, and neutral body postures. (**B**) Trial example.

Different images were displayed on a 15.4-inch screen positioned approximately 60 cm from the participants. A total of 48 pictures depicting four different actors (two males and two females) in both emotional and neutral poses (Fig. 1.a) were chosen from a validated database^19,21,38^. These images were equally categorized into 16 instances of fearful postures, 16 of happy postures, and 16 of neutral body movements (Fig. 1). The emotional expressions were elicited through emotionally engaging instructions aimed at generating spontaneous and naturalistic reactions, rather than posed gestures. Images were then selected based on pilot studies assessing emotional intensity and recognition accuracy, ensuring the inclusion of stimuli with high emotional clarity and minimal ambiguity. For details see^38^.

### Transcranial magnetic stimulation and electromyography recording

The experiment began with setting up the electrode montage, identifying the optimal scalp position, and determining the resting motor threshold (rMT). To assess CSE, we elicited MEPs by stimulating the hand M1 representation in the left and right hemispheres. Specifically, we targeted the motor representation of the first dorsal interosseous (FDI), using a Magstim 200 transcranial magnetic stimulator (Magstim, Whiteland, Dyfed, UK) equipped with a 70-mm figure-of-eight focal coil. Electromyography (EMG) signals from both the right and left FDI were recorded with a Biopac MP-35 device (Biopac, U.S.A.), band-pass filtered from 30 to 500 Hz, sampled at 5000 Hz, digitized, and stored for offline analysis. Pairs of silver-chloride surface electrodes were positioned in a belly-tendon configuration over the FDI of the two hands, with ground electrodes placed on the wrist. The coil was held tangentially to the scalp, with its handle oriented backward and laterally at a 45° angle from the midline to generate a posterior-anterior current flow across the central sulcus. The optimal coil position was defined as the M1 site where stimulation consistently produced the largest MEP in the contralateral FDI. The rMT was defined as the minimum stimulus intensity needed to evoke MEPs of at least 50 µV in 5 out of 10 consecutive pulses^40^. TMS was applied at 120% of the rMT across two sessions, each involving a different behavioral task (see below). Within each session, two blocks were performed, with stimulation applied to the left M1 in one block and to the right M1 in the other. Mean stimulation intensity (mean % of maximal stimulator output ± SD) was statistically comparable between the right M1 block (57.68 ± 8.09) and left M1 block (57.77 ± 8.14; t_42_ = 0.04, *p* = 0.97). We consistently monitored for any EMG muscle contractions, and their absence was visually confirmed during the entire experiment.

### Procedure and experimental design

The experiments were programmed using Matlab software, which controlled the presentation of the images and the triggering of TMS pulses. In the first TMS session (main experiment), we assessed CSE during the presentation of visual stimuli. MEPs induced by stimulation of the left and right M1 were recorded in two separate experimental blocks of 144 trials each (Fig. 1.b), with M1 stimulation applied to the left M1 in one block and to the right M1 in the other. The order of the two blocks was counterbalanced across participants. Participants performed a sex discrimination task: a neutral body picture was presented as the target stimulus, and participants had to decide whether the depicted model was male or female. Target stimuli were preceded by the brief presentation of prime stimuli, following this procedure: each trial began with a gray screen displayed for 1 s, followed by a random-dot mask lasting 16 ms. This mask was generated by scrambling the corresponding sample stimulus using custom-made image segmentation software. Subsequently, the prime stimulus —depicting a happy, fearful, or neutral body posture— was presented at the center of the random-dot mask for 16 ms. This was immediately followed by another mask lasting 33 ms and then the target stimulus, which always depicted a neutral body posture. For each prime-target pair, the same model was depicted in both the prime and target stimuli to ensure consistency in body sex and actor identity.

Participants were instructed to perform the sex discrimination task on the target stimulus by providing a verbal response, which was recorded by an experimenter pressing a key on the computer. To avoid altering CSE due to verbal response^41,42^, participants were told to respond a few seconds after the TMS pulse, during the presentation of the question screen^43^. After their response, a white screen was presented for 6 s. In this way, we ensured a low TMS frequency, in order to prevent CSE changes caused by TMS itself^44^ throughout the experiment. The target stimuli were presented for 80, 100, or 120 ms and TMS pulses were delivered at 70, 90, and 110 ms from stimulus onset. Stimulus duration was randomly distributed across each block.

Following the first TMS session (main experiment), we performed two awareness checks. The first awareness check (subjective) was carried out without TMS and aimed to assess participants’ subjective awareness of the prime body posture. Participants were asked a series of questions in the following order: (1) Did you see anything besides the target body? (2) Did you see anything right before the target body? (3) There was actually a flicker before each target body: what did you see? (4) Did you see a body? (5) What expression did you see? Verbal reports indicating awareness of a body and/or expression were considered evidence that the prime presentations were not strictly subliminal^32,45^. Next, participants were debriefed about the use of subliminal primes.

The second awareness check (objective) was designed to assess participants’ objective awareness of the prime body stimuli while receiving TMS pulses over M1. The procedure mirrored the timing and structure of the main experiment: TMS pulses were delivered over the two M1 to simulate the same potential neural interference of TMS present during CSE measurement. However, no MEPs were recorded, as participants were asked to discriminate the prime stimulus by providing a manual response (forced choices: happy, fearful, neutral). Participants provided their answers to the emotion discrimination task by pressing a computer key a few seconds after the TMS pulse. On each trial, participants also judged their confidence on a 4-point Likert scale. Information about participants’ awareness and confidence was collected in two separate blocks of 48 trials, one for each hemisphere.

### Subjective measures: personality questionnaires

In our previous study^18^, we observed that CSE reduction when observing fearful body postures was associated with interindividual differences in behavioral inhibition system (BIS) scale, while behavioral activation system (BAS) scale or State and Trait-Anxiety Inventory (STAI-Y2) showed no consistent predictive value. Therefore, to assess whether similar inter-individual differences may also predict CSE modulation for unconscious fearful body expressions presentation, after the two TMS sessions, we asked participants to fill out the Italian versions of the BIS/BAS^46,47^, the form Y2 of the STAI^48^. The BIS/BAS scales are based on the idea that two primary motivational systems influence behavior. The BIS is responsible for regulating responses to potential threats, promoting caution, fear, and anxiety when faced with danger. In contrast, the BAS drives approach behaviors, motivating individuals to pursue rewards and positive outcomes, and to avoid punishment. The BIS scale, which includes 7 items, measures an individual’s sensitivity to threats, while the BAS scale, consisting of 13 items, assesses the tendency to seek out rewards and pleasurable experiences. On the other hand, the STAI-Y2 is a general anxiety questionnaire that measures how frequently a person experiences anxiety. Unlike the BIS/BAS scales, which focus on responses to specific threats and rewards, the STAI-Y2 provides a broader assessment of overall anxiety levels, measuring the general tendency toward anxiety rather than specific triggers.

### Data analysis

Neurophysiological and behavioral data were processed off-line. The mean MEP amplitude in each condition was measured peak-to-peak (in mV). MEPs associated with incorrect answers were discarded from the analysis (11%), in line with high task accuracy (89% ± 31%). Since background EMG is known to affect motor excitability^49^MEPs with preceding background EMG deviating from the mean by more than 2 S.D. were removed from further analysis (~ 3%).

MEP amplitudes were first analyzed using a four-way ANOVA with Hemisphere (2 levels: Right and Left), Time (3 levels: 70, 90 and 110 ms), actor Sex (2 levels: Female, Male), and Prime Body Expression (3 levels: Happy, Fearful and Neutral) as within-subjects factors. Post-hoc comparisons were carried out by means of the Newman-Keuls test. Effect size indices for main effects and interactions were computed using *partial eta*^2^, whereas *Cohen’s d* was computed for post hoc comparisons.

The ANOVAs showed a CSE reduction for the prime showing fearful body postures relative to happy and neutral prime body postures in the left hemisphere, and this effect was independent of the factor Time. To explore the relations between this motor suppression and key personality traits^18^correlation and regression analyses were performed. An index representing the early motor modulation [the mean CSE effect for fearful postures in the left hemisphere (average across time and actor sex) minus the mean CSE of all the other conditions, was entered as the dependent variable in a stepwise regression model, whereas questionnaire scores were entered as predictors (criteria probability of *F*-to-enter: ≤ 0.05; *F*-to-remove: ≥0.1).

Moreover, to account for participants’ objective awareness of subliminal primes, mean scores of accuracy collected during the two control experiments (i.e., TMS over right or left M1) were entered in a two-way ANOVA with Hemisphere (2 levels: Right and Left) x Prime Body Expression (3 levels: Happy, Fearful and Neutral) as within-subjects factors. This analysis tested whether the MEP modulation observed in the main experiment (see Results) could be attributed to specific interferential effects of TMS on the discrimination of prime stimuli.

Since the mean accuracy data exceeded the chance level (33%), we conducted additional analyses to determine whether subjective awareness influenced CSE modulation. Participants were classified as either ‘aware’ or ‘unaware’ of the prime based on individual performance assessed using two-sided binomial tests. Those with accuracy significantly above chance (*p* < 0.05) were categorized as ‘aware’, while the rest were classified as ‘unaware’. Then, to investigate whether the reduction of CSE was influenced by interindividual differences in awareness, a mixed factors 3-way ANOVA was performed on MEP amplitudes with Awareness (2 levels: Aware, Unaware) as the between-subjects factor and Hemisphere and Prime Body Expression as within-subjects factors. The same ANOVA was calculated on a subsample of MEP selecting the first 48 stimuli presentation (i.e., 16 body postures for each body expression) for each hemisphere to have a comparable number of measurements in the main experiment (i.e., during the sex discrimination task on the target stimulus) compared to the control experiment (i.e., during the emotion discrimination task on the prime stimulus).

To assess participants’ metacognitive sensitivity, we compared meta-d’ to d’ scores, with the target signal being fear, and noise conditions represented by other prime body expressions (e.g., neutral or happy). Meta-d’ was calculated specifically for trials where participants reported high confidence, defined as the top 50% of confidence scores (those rated ≥ 3 on a 1 to 4 scale). This approach allowed us to focus on trials where participants felt subjectively certain of their responses, thereby providing a clearer measure of metacognitive sensitivity in relation to actual detection ability. A paired samples *t*-test was conducted to examine potential differences between meta-d’ and d’ scores, revealing the degree to which participants’ confidence corresponded to their detection accuracy. Additionally, we analyzed criteria for both meta-d’ and d’ scores to assess response bias across conditions, helping to determine whether participants maintained consistent decision thresholds regardless of confidence level.

To further explore the relationship between metacognitive sensitivity and personality traits, correlation analyses were performed between meta-d’ and scores from the BIS, BAS, and STAI-Y2. This was done to evaluate whether individual differences in these traits might predict variations in metacognitive sensitivity. Finally, to assess whether participants’ level of metacognitive sensitivity influenced CSE modulation, we used meta-d’ as a criterion to split participants into two groups: those with high metacognitive sensitivity (meta-d’ > 1) and those with low metacognitive sensitivity (meta-d’ ≤ 1). This allowed us to examine whether variations in metacognitive sensitivity were associated with differential effects on CSE. A mixed-factor ANOVA was conducted across metacognitive sensitivity levels, Hemispheres, and different Prime Body Expressions on MEP amplitude.

For all ANOVAs, effect size indices for main effects and interactions were computed using *partial eta*^2^ (*η*_*p*_^2^, whereas *Cohen’s d* was computed for post hoc comparisons.

## Results

### Neurophysiological data

**Fig. 2:**
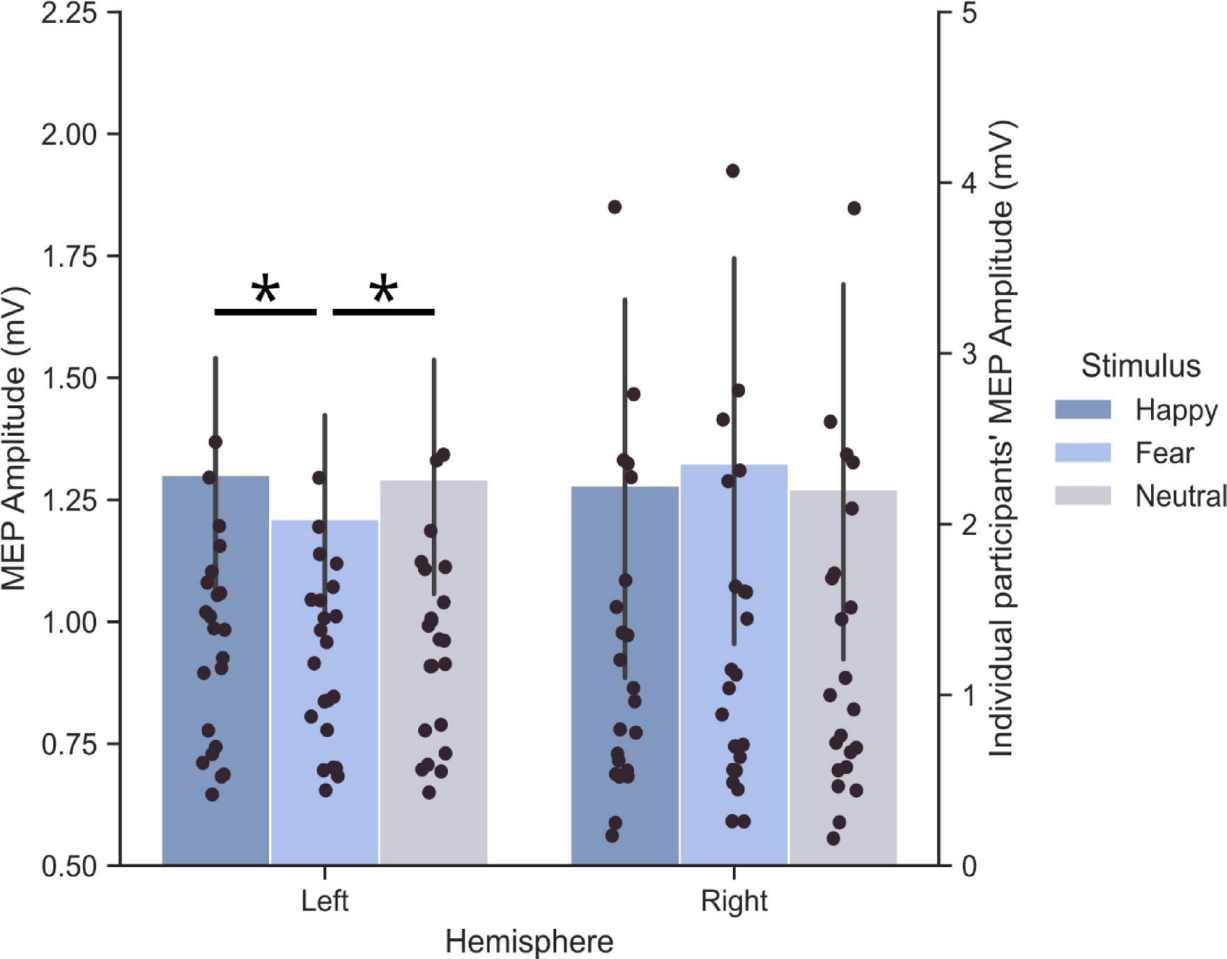
CSE modulations during the sex discrimination task. MEP amplitude (mV) was recorded from FDI muscle. Data show the Hemisphere × Body Posture interaction (average of the three Times and Sex). Asterisks (*) denote significant post-hoc comparisons (*p* < 0.05). Error bars indicate s.e.m. Different scales were used for mean (on the left) and individual data (on the right).

The Hemisphere × Time × Sex × Prime Body Expression ANOVA on MEP amplitudes showed a significant Hemisphere × Prime Body Expression interaction (*F*_2,42_ = 7.88; *p* = 0.001; *η*_*p*_^*2*^ = 0.27; Fig. 2). Post-hoc analyses showed that lower MEPs were elicited in the left hemisphere when participants observed fearful postures (mean amplitude ± S.D. = 1.21 ± 0.11 mV) rather than happy (1.30 ± 0.12 mV, *p* = 0.01; *d* = 0.76) and neutral expressions (1.29 ± 0.12 mV, *p* = 0.02; *d* = 0.69), which in turn did not significantly differ from one another (*p* = 0.73; *d* = 0.08). Moreover, MEP amplitudes recorded for fearful postures in the left hemisphere were lower than fearful (1.32 ± 0.21 mV, *p* = 0.002, *d* = 0.68), happy (1.28 ± 0.20 mV, *p* = 0.04; d = 0.42), and neutral postures (1.27 ± 0.19 mV, *p* = 0.04; *d* = 0.37) recorded in the right hemisphere. No significant modulations were found in the right hemisphere (all *p* ≥ 0.31). In sum, the analysis indicated that body expressions modulated MEPs only in the left hemisphere session, with lower amplitudes for fearful relative to happy and neutral bodies, but not in the right hemisphere. No other main effects or interactions were significant (all *F* ≤ 2.42, *p* ≥ 0.10; all *η*_*p*_^*2*^ ≤ 0.0007). In particular, the Hemisphere × Prime Body interaction was not qualified by higher-order interactions with either Time or Sex (0.43 ≤ *F* ≤ 2.42, 0.78 ≥ *p* ≥ 0.10), suggesting that the left-M1 CSE reduction for fearful postures did not vary as function of stimulation latencies and sex of the actors.

### Relation between changes in motor excitability and personality traits

The reduction in CSE for observed fearful body expressions in the left M1 was found across all time intervals. A series of correlations and a multiple regression analysis were carried out to test whether this neurophysiological effect was related to individual differences in affective personality traits. A MEP contrast computed based on the results of the ANOVA (i.e., [the mean CSE modulation for fearful postures in the left hemisphere (average across time and sex) minus the mean CSE of all the other conditions was entered into the analysis as a dependent variable, and participants’ scores from the BIS, BAS, and STAI-Y2 scales were entered as predictors. The regression model and simple correlations between the magnitude of CSE suppression and BIS, BAS, and STAI-Y2 scores were not significant (− 0.02 ≤ *r* ≤ 0.41, all *p* ≥ 0.09). Thus, the suppression of the CSE in response to unconscious fearful body postures was not significantly associated with personality variables in our sample, unlike what has been observed with consciously perceived stimuli^18^.

### Subjective awareness check

Out of 22 participants, four explicitly reported the presence of the body postures as primes. To ensure that the results were not influenced by this factor, we repeated the same Hemisphere × Time × Sex × Prime Body Expression ANOVA after excluding these four participants. The results confirmed the significant Hemisphere × Prime Body Expression interaction (*F*_2,34_ = 7.43, *p* = 0.002, *η*_*p*_^*2*^ = 0.30). This suggests that the fear-specific modulations are detectable even when the prime stimuli are not consciously perceived, indicating that subjective prime visibility is not crucial for eliciting CSE suppression.

### Objective awareness check

The Hemisphere × Prime Body Expression ANOVA conducted on the accuracy scores collected during the emotion discrimination task showed no significant main effects of Hemisphere (*F*_1,21_ = 0.88; *p* = 0.36; *η*_*p*_^2^ = 0.04) or Prime Body Expression (*F*_2,42_ = 0.43; *p* = 0.65; *η*_*p*_^2^ = 0.02), and no significant Hemisphere × Prime Body Expression interaction (*F*_2,42_ = 0.07; *p* = 0.93; *η*_*p*_^2^ = 0.003), indicating that neither active stimulation of the two hemispheres nor the presentation of different body postures significantly impacted perceptual awareness in our sample (see Table 1).

**Table 1:** Mean ± SD of the accuracy in the emotion discrimination task shown separately for the two hemispheres (i.e., TMS over right or left M1).

	Left hemisphere	Right hemisphere
Happy body posture	46% ± 22	44% ± 20
Fearful body posture	43% ± 17	41% ± 17
Neutral body posture	46% ± 17	46% ± 14

The binomial test was conducted to categorize participants as ‘aware’ and ‘unaware’. Half of the subjects were able to discriminate above chance the presented body postures (mean accuracy scores for 11 participants out of 22 were higher than 33% (*p* ≤ 0.05), whereas the remaining participants were unable to discriminate postures above chance level. Then, the Awareness × Hemisphere × Prime Body Expression on MEP amplitude mixed factors ANOVA revealed no main effect of Awareness (*F*_1,20_ = 0.16, *p* = 0.69; *η*_*p*_^2^ = 0.0001), and no significant interactions involving Awareness (all *F* ≤ 0.97; all *p* ≥ 0.38). We can therefore conclude that the effect we found in the CSE (i.e., reduction in the CSE for fearful relative to happy and neutral body postures in the left hemisphere) did not significantly differ across aware and unaware participants.

**Fig. 3:**
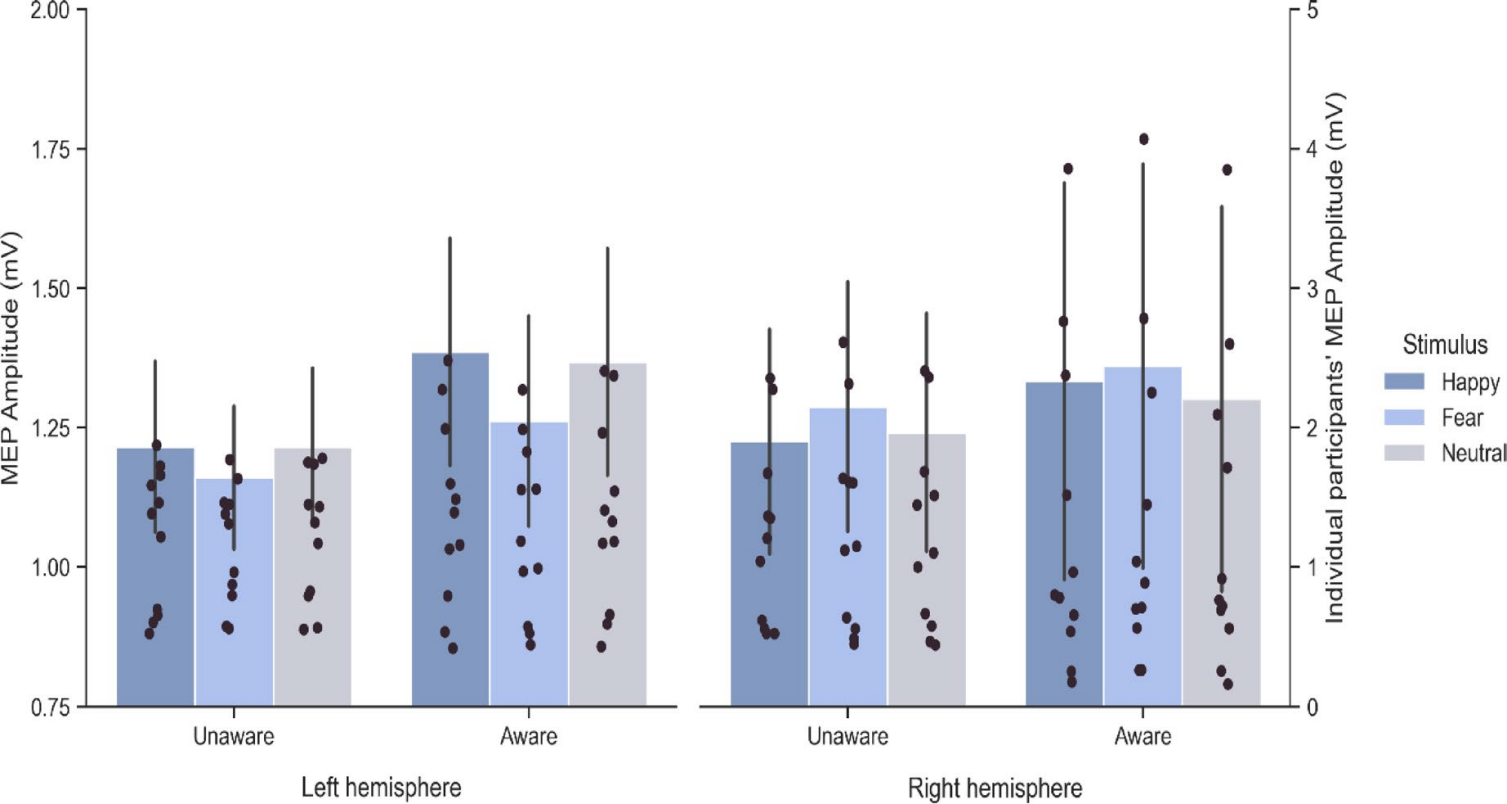
Awareness effect on CSE modulations during the sex discrimination task. MEP amplitude (mV) was recorded from FDI muscle. Participants were classified as aware or unaware according to their accuracy scores in the emotion discrimination task and the binomial test. Data show the Awareness × Hemisphere × Prime Body Expression interaction. Error bars indicate s.e.m. Different scales were used for mean (on the left) and individual data (on the right).

The same Awareness × Hemisphere × Prime Body Posture mixed factors ANOVA was repeated on a subsample of MEP (the first 48 presented stimuli, see method session) and results showed no main effect of the factor Awareness (*F*_1,20_ = 0.28; *p* = 0.60; *η*_*p*_^*2*^ = 0.01) and no significant interactions (all *F* ≤ 0.46; all *p* ≥ 0.63). These results additionally support the idea that prime visibility did not consistently influence CSE suppression in our experiment (Fig. 3).

### Confidence analysis

**Fig. 4:**
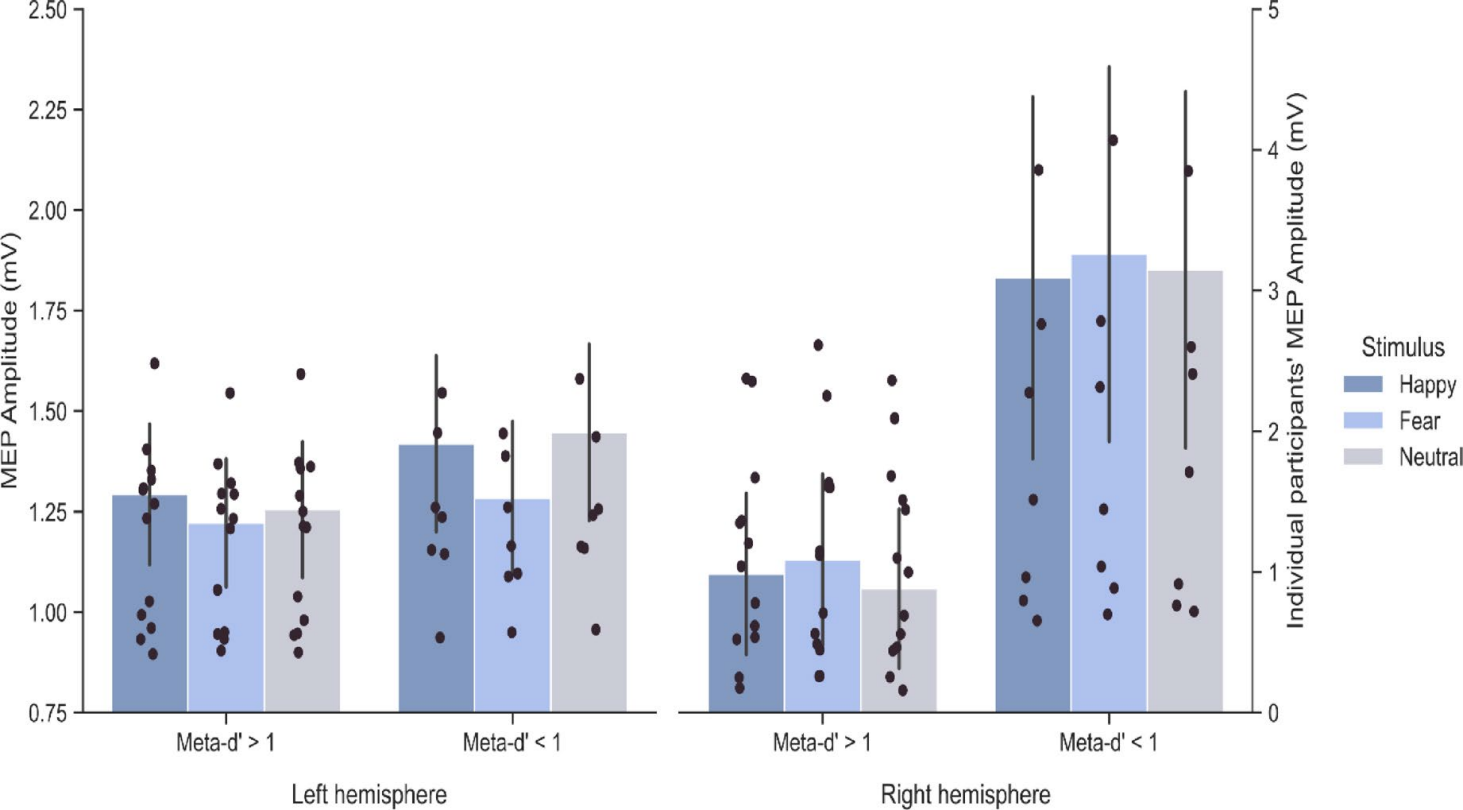
Comparison of CSE modulations during the sex discrimination task based on meta-d’ scores. Participants were classified into two groups: Meta-d’ > 1 and Meta-d’ < 1. Corticospinal excitability (CSE) was assessed by recording motor-evoked potential (MEP) amplitude (mV) from the FDI muscle. The data illustrate the interaction between meta-d’ scores and Hemisphere on MEP amplitude. Error bars represent the standard error of the mean (s.e.m.).

A paired samples *t*-test revealed a significant difference between d’ and meta-d’, indicating that participants’ metacognitive sensitivity was higher (meta-d’ mean ± S.D. = 0.97 ± 0.91) than their objective detection ability (d’ mean ± S.D. = 0.49 ± 0.56; *t*_19_ = 3.07, *p* = 0.006, *d* = 0.64). This suggests a moderate effect size, with participants showing greater alignment between confidence and accuracy on high-confidence trials. Additionally, there was no significant difference in criterion values between meta criterion (mean ± S.D. = 0.39 ± 0.36) and criterion (mean ± S.D. = 0.45 ± 0.23), indicating that participants did not significantly change their response bias across conditions, regardless of confidence level (*t*_19_ = -0.68, *p* = 0.50, *d* = 0.18). These findings suggest that participants could accurately gauge their performance on trials where they felt more confident, reflected in a higher meta-d’ compared to d’. However, their criterion for emotion discrimination remained consistent across both high-confidence and general trials, demonstrating no significant shift in response bias. Simple correlations between the magnitude of meta-d’ and BIS, BAS, and STAI-Y2 scores were not significant (-0.27 ≤ *r* ≤ 0.12, all *p* ≥ 0.24). Then, the Meta-d’ × Hemisphere × Prime Body Expression on MEP amplitude mixed factors ANOVA revealed no main effect of the factor Meta-d’ (*F*_1,18_ = 2.35, *p* = 0.14; *η*_*p*_^2^ = 0.11) and no significant interactions involving Meta-d’ were found (all *F* < 0.75; all *p* > 0.12; Fig. 4). Thus, we observed no influence of participants’ metacognitive sensitivity in the effect we found in the CSE (i.e., reduction in the CSE for fearful relative to happy and neutral body postures in the left hemisphere).

## Discussion

The human brain rapidly and efficiently processes significant environmental information, even when it lies outside our attention or awareness. Previous EEG studies have shown that early visual components (e.g., P1, N170, and P200) are influenced by the subliminal presentation of fearful stimuli^50–57^. However, whether the motor system is similarly affected at the early stages of processing remains unclear. To address this issue, we tested CSE in the left (dominant) and right M1 of healthy participants performing a sex discrimination task on neutral body postures. Subliminal task-irrelevant body postures (negative, positive, or neutral) were flashed before the target neutral stimuli, and CSE was probed at early latencies (70, 90, and 110 ms) post-stimulus onset^18,19,21,23^. Additionally, participants’ personality traits, subjective and objective awareness of stimulus detection, and metacognitive sensitivity (i.e., the ability to gauge one’s performance accurately) were considered potential modulators^58,59^.

We found that subliminally presented fearful body postures reduced CSE in the left, dominant hemisphere (i.e., lower MEP in the right hand). This supports our hypothesis that task-irrelevant subliminal fearful stimuli modulate the motor system at early processing stages, likely preceding conscious visual awareness. As expected, this CSE suppression was independent of stimulation timing, consistent with prior findings of CSE reduction in response to supraliminal fearful postures at similar early time windows^18,19,21,22^.

Importantly, the modulation was lateralized to the left M1, highlighting the engagement of dominant-hemisphere circuits involved in action planning, inhibitory control, and attentional processes (e.g^60,61^. This lateralization is particularly relevant considering that all participants in our study were right-handed, the left hemisphere is typically dominant for motor control in such individuals^62,63^. Previous studies have suggested that left M1 shows greater CSE modulation during tasks requiring rapid motor responses, suggesting a functional asymmetry that facilitates fast action execution^64,65^, including increased readiness to emotional stimuli^66^.

Left-lateralized modulation may therefore indicate that early motor inhibition processes are preferentially initiated in the dominant hemisphere, serving as an initial gating mechanism. Engaging early inhibitory control in the dominant hemisphere may allow for fast and efficient modulation of motor output, given its leading role in voluntary movement and action planning in right-handed individuals. However, we do not interpret this effect as hand-specific motor inhibition. Rather, we suggest that the left M1 may serve as a functional entry point for a broader motor suppression cascade, rapidly recruiting bilateral networks to support a transient, global freezing-like response—consistent with previous findings of widespread bilateral inhibition at slightly later latencies (100–125 ms^22,31^. In real-life conditions, such transient early inhibition may serve an adaptive function: a brief suppression of motor output may act as a preparatory step that prevents premature or maladaptive actions, enhances vigilance, and allows for rapid contextual assessment^24–27^. This pause would then facilitate the timely recruitment of broader, bilateral motor programs required for an effective full-body defensive response.

Interestingly, in our recent work, CSE suppression in the left M1 was observed when participants actively monitored emotional features of supraliminal body postures during an emotion recognition task; in contrast, the same stimuli elicited CSE facilitation when participants performed a gender recognition task, despite involving the same emotional body postures^21^. These earlier findings suggest that motor inhibition during conscious perception of emotional body postures is modulated by task demands and emerges specifically when attention is directed toward emotional features. In contrast, here, fearful stimuli were presented subliminally and were task-irrelevant, yet still elicited a reduction in CSE. This provides compelling evidence that threat-related motor suppression can occur automatically, without conscious awareness or explicit attentional engagement. Such automatic inhibition likely reflects an early, preattentive defensive mechanism—such as freezing—that prepares the organism for rapid and adaptive responses to potential danger^24–27^.

Our study provides the first evidence of the impact of subliminal body expressions on the observer’s CSE. This contrasts with Engelen et al.^33^, who reported null findings when using continuous flash suppression (CFS) paradigms and later stimulation intervals (200–700 ms post-stimulus). The discrepancy may stem from differences in timing and paradigms, as our study targeted early time windows (70–100 ms), while later intervals capture more general action-related effects^22,38^. Furthermore, the gradual contrast increase inherent to CFS might attenuate early perceptual processing, thereby limiting the stimulus’s impact on MEP amplitude.

Our findings suggest that CSE suppression was independent of participants’ subjective or objective awareness of prime stimuli, as well as metacognitive sensitivity. Excluding participants reporting subjective awareness did not alter the observed CSE suppression. Additionally, metacognitive sensitivity—the confidence-accuracy relationship—was unrelated to CSE modulation or personality traits, reinforcing the role of unconscious mechanisms in motor system responses to subliminal fearful cues.

Unlike previous studies linking personality traits, particularly BIS-related traits, to heightened sensitivity to fear-related stimuli under conscious conditions^18,22^, we found no such association under subliminal conditions in our study. Although this should be interpreted with caution—given that a lack of statistical significance does not imply the absence of an effect, especially with a relatively small sample—the dissociation between the present and prior^18,22^ findings may suggest that the motor system’s response to subliminal fear operates independently of individual personality differences. With these caveats, our results appear to support the “low-road” pathway hypothesis^67^, which emphasizes rapid subcortical processing via structures such as the pulvinar, caudate nucleus, and amygdala^12,68,69^. This pathway enables preparatory motor responses, such as freezing or avoidance, bypassing slower cortical processing. Subcortical structures also project downstream to influence spinal cord responses^70,71^. In contrast, consciously perceived fear engages the “high road,” integrating personal experiences and contextual factors, and is more influenced by traits like BIS^72,73^.

This study highlights the motor system’s rapid, automatic response to subliminal threats, likely mediated by an evolutionary conserved mechanism for survival. The observed independence from personality traits and subjective/objective awareness further underscores its automatic nature. Further research is needed to elucidate the neurophysiological underpinnings and functional or clinical implications of these motor responses. Additionally, exploring the temporal dynamics and hemispheric specialization of subliminal threat processing could refine our understanding of these mechanisms. For example, the structural and functional asymmetry of the left M1, including its greater volume of descending motor fibers, may explain its role in facilitating swift motor responses to emotional stimuli^74,75^.

In conclusion, this work provides compelling evidence that the motor system can rapidly and selectively respond to subliminal fearful cues. These findings emphasize the importance of temporal precision and subcortical mechanisms in understanding the interplay between emotion and motor control, offering novel insights into the rapid and unconscious preparation of defensive actions in response to perceived threats.

## Supplementary Information

Below is the link to the electronic supplementary material.

Supplementary Material 1

## Data Availability

The datasets generated during this study are available at Open Science Framework Repository (https://osf.io/vsu72/?view_only=32eb5091bf9f485eaf035cf42105b8c2).
