# Supplementary material for: Early changes in corticospinal excitability for subliminally presented fearful body postures
Source: Sci Rep. 2025 Aug 8;15:29088. doi: 10.1038/s41598-025-13185-y (PMC12334645; doi:10.1038/s41598-025-13185-y)
Supplement: Supplementary file 1 — Supplementary Material 1 [file 41598_2025_13185_MOESM1_ESM.docx]

This supplementary material provides the full statistical analyses reported in the main manuscript:

**Early changes in corticospinal excitability for subliminally presented fearful body postures**

**Neurophysiological data**

ANOVA on MEP Amplitudes

Model: Hemisphere × Time × Sex × Prime Body Expression

|  |  |  |  |  |  |
| --- | --- | --- | --- | --- | --- |
|  | SS | DF | MS | F | P |
| HEMISPHERE | 0.114 | 1 | 0.114 | 0.016 | 0.901 |
| TIME | 0.265 | 2 | 0.133 | 1.064 | 0.354 |
| SEX | 0.014 | 1 | 0.014 | 0.171 | 0.683 |
| PRIME | 0.066 | 2 | 0.033 | 0.701 | 0.502 |
| HEMISPHERE*TIME | 0.116 | 2 | 0.058 | 1.077 | 0.35 |
| HEMISPHERE*SEX | 0.071 | 1 | 0.071 | 1.729 | 0.203 |
| TIME*SEX | 0.062 | 2 | 0.031 | 0.442 | 0.646 |
| HEMISPHERE*PRIME | 0.792 | 2 | 0.396 | 7.884 | 0.001 |
| TIME*PRIME | 0.145 | 4 | 0.036 | 0.384 | 0.82 |
| SEX*PRIME | 0.01 | 2 | 0.005 | 0.063 | 0.939 |
| HEMISPHERE*TIME*SEX | 0.162 | 2 | 0.081 | 0.866 | 0.428 |
| HEMISPHERE*TIME*PRIME | 0.201 | 4 | 0.05 | 0.787 | 0.537 |
| HEMISPHERE*SEX*PRIME | 0.454 | 2 | 0.227 | 2.421 | 0.101 |
| TIME*SEX*PRIME | 0.143 | 4 | 0.036 | 0.434 | 0.783 |
| HEMISPHERE*TIME*SEX*PRIME | 0.64 | 4 | 0.16 | 2.397 | 0.057 |

Pairwise Comparisons for Time * Prime Interaction

|  |  |  |  |  |  |  |  |
| --- | --- | --- | --- | --- | --- | --- | --- |
|  |  | Left | Left | Left | Right | Right | Right |
|  |  | Happy | Fear | Neutral | Happy | Fear | Neutral |
| Left | Happy |  | 0.018 | 0.733 | 0.717 | 0.395 | 0.310 |
| Left | Fear |  |  | 0.028 | 0.045 | 0.002 | 0.036 |
| Left | Neutral |  |  |  | 0.664 | 0.458 | 0.750 |
| Right | Happy |  |  |  |  | 0.368 | 0.775 |
| Right | Fear |  |  |  |  |  | 0.319 |
| Right | Neutral |  |  |  |  |  |  |

# **Relation between changes in motor excitability and personality traits**

ANOVA on MEP contrasts

Model: BIS + BAS + STAI-Y2

|  | SS | DF | MS | F | p |
| --- | --- | --- | --- | --- | --- |
| STAI-Y2 | 0.006 | 1 | 0.006 | 0.02 | 0.887 |
| BIS | 0.617 | 1 | 0.617 | 2.106 | 0.164 |
| BAS | 0.015 | 1 | 0.015 | 0.05 | 0.825 |
|  |  |  |  |  |  |

Correlation matrix (n = 22)

|  | STAI-Y2 | BIS | BAS | MEP contrasts |
| --- | --- | --- | --- | --- |
| STAI-Y2 |  | 0.6143 | -0.3386 | 0.1883 |
|  |  | p = 0.002 | p = 0.123 | p = 0.401 |
| BIS |  |  | -0.2102 | 0.4161 |
|  |  |  | p = 0.348 | p = 0.094 |
| BAS |  |  |  | -0.0185 |
|  |  |  |  | p = 0.935 |
| MEP contrasts |  |  |  |  |
|  |  |  |  |  |

# **Subjective Awareness check**

ANOVA on MEP amplitudes

Model: Hemisphere × Time × Sex × Prime Body Expression

|  | SS | | DF | MS | F | p |
| --- | --- | --- | --- | --- | --- | --- |
| HEMISPHERE | | 0.236 | 1 | 0.236 | 0.039 | 0.845 |
| TIME | | 0.383 | 2 | 0.191 | 1.558 | 0.225 |
| SEX | | 0.013 | 1 | 0.013 | 0.14 | 0.713 |
| PRIME | | 0.07 | 2 | 0.035 | 0.63 | 0.539 |
| HEMISPHERE*TIME | | 0.071 | 2 | 0.035 | 0.581 | 0.565 |
| HEMISPHERE*SEX | | 0.03 | 1 | 0.03 | 0.738 | 0.402 |
| TIME*SEX | | 0.051 | 2 | 0.025 | 0.344 | 0.712 |
| HEMISPHERE*PRIME | | 0.681 | 2 | 0.341 | 7.425 | 0.002 |
| TIME*PRIME | | 0.375 | 4 | 0.094 | 1.265 | 0.292 |
| SEX*PRIME | | 0.02 | 2 | 0.01 | 0.118 | 0.889 |
| HEMISPHERE*TIME*SEX | | 0.049 | 2 | 0.025 | 0.257 | 0.775 |
| HEMISPHERE*TIME*PRIME | | 0.154 | 4 | 0.038 | 0.548 | 0.701 |
| HEMISPHERE*SEX*PRIME | | 0.431 | 2 | 0.216 | 2.094 | 0.139 |
| TIME*SEX*PRIME | | 0.28 | 4 | 0.07 | 1.019 | 0.404 |
| HEMISPHERE*TIME*SEX*PRIME | | 0.402 | 4 | 0.1 | 1.33 | 0.268 |

# **Objective Awareness check**

ANOVA on Accuracy

Model: Hemisphere × Time × Sex × Prime Body Expression

|  | SS | DF | MS | F | p |
| --- | --- | --- | --- | --- | --- |
| HEMISPHERE | 0.005 | 1 | 0.005 | 0.884 | 0.368 |
| PRIME | 0.034 | 2 | 0.017 | 0.434 | 0.654 |
| HEMISPHERE*PRIME | 0.002 | 2 | 0.001 | 0.073 | 0.93 |

Binomial test

| id | correct | N responses | p |
| --- | --- | --- | --- |
| 1 | 61 | 144 | 0.0267 |
| 2 | 47 | 144 | 0.9296 |
| 3 | 54 | 144 | 0.2898 |
| 4 | 46 | 144 | 0.7910 |
| 5 | 56 | 144 | 0.1582 |
| 6 | 50 | 144 | 0.7242 |
| 7 | 52 | 144 | 0.4804 |
| 8 | 46 | 144 | 0.7910 |
| 9 | 52 | 144 | 0.4804 |
| 10 | 58 | 144 | 0.0926 |
| 11 | 78 | 144 | 0.0000 |
| 12 | 49 | 144 | 0.8600 |
| 13 | 51 | 144 | 0.5966 |
| 14 | 72 | 144 | 0.0000 |
| 15 | 63 | 144 | 0.0101 |
| 16 | 101 | 144 | 0.0000 |
| 17 | 60 | 144 | 0.0416 |
| 18 | 72 | 144 | 0.0000 |
| 19 | 84 | 144 | 0.0000 |
| 20 | 108 | 144 | 0.0000 |
| 21 | 79 | 144 | 0.0000 |
| 22 | 68 | 144 | 0.0007 |

ANOVA on MEP

Model: Awareness × Hemisphere × Prime Body

|  | SS | DF | MS | F | p |
| --- | --- | --- | --- | --- | --- |
| Awareness | 0.41 | 1 | 0.41 | 0.164 | 0.69 |
| HEMISPHERE | 0.019 | 1 | 0.019 | 0.015 | 0.903 |
| HEMISPHERE*Awareness | 0.031 | 1 | 0.031 | 0.025 | 0.877 |
| PRIME | 0.011 | 2 | 0.006 | 0.722 | 0.492 |
| PRIME*Awareness | 0.016 | 2 | 0.008 | 0.969 | 0.37 |
| HEMISPHERE*PRIME | 0.135 | 2 | 0.068 | 7.888 | 0.001 |
| HEMISPHERE*PRIME*Awareness | 0.005 | 2 | 0.003 | 0.297 | 0.745 |

ANOVA on MEP (first 48 stimuli)

Model: Awareness × Hemisphere × Prime Body

|  | SS | DF | MS | F | p |
| --- | --- | --- | --- | --- | --- |
| Awareness | 0.697 | 1 | 0.697 | 0.283 | 0.601 |
| HEMISPHERE | 0.212 | 1 | 0.212 | 0.194 | 0.664 |
| HEMISPHERE*Awareness | 0.02 | 1 | 0.02 | 0.018 | 0.893 |
| PRIME | 0.004 | 2 | 0.002 | 0.087 | 0.917 |
| PRIME*Awareness | 0.06 | 2 | 0.03 | 0.465 | 0.631 |
| HEMISPHERE*PRIME | 0.308 | 2 | 0.154 | 4.927 | 0.012 |
| HEMISPHERE*PRIME*Awareness | 0.003 | 2 | 0.002 | 0.049 | 0.952 |

# **Confidence analysis**

All t-tests are reported in the main manuscript.

Correlation matrix (n = 20)

|  | meta-d' | STAI-Y2 | BIS | BAS |
| --- | --- | --- | --- | --- |
| meta-d' |  | 0.1222 | 0.0829 | -0.0683 |
|  |  | p = 0.608 | p = 0.728 | p = 0.775 |
| STAI-Y2 |  |  | 0.6046 | -0.27 |
|  |  |  | p = 0.005 | p = 0.250 |
| BIS |  |  |  | -0.1864 |
|  |  |  |  | p = 0.431 |
| BAS |  |  |  |  |

ANOVA on MEP
Model: Meta-d’ × Hemisphere × Prime Body Expression

|  | SS | | DF | MS | F | p |
| --- | --- | --- | --- | --- | --- | --- |
| meta-d' | | 5.403 | 1 | 5.403 | 2.351 | 0.143 |
| HEMISPHERE | | 0.667 | 1 | 0.667 | 0.551 | 0.467 |
| HEMISPHERE* meta-d' | | 2.771 | 1 | 2.771 | 2.289 | 0.148 |
| PRIME | | 0.015 | 2 | 0.008 | 1.033 | 0.366 |
| PRIME* meta-d' | | 0.033 | 2 | 0.017 | 2.215 | 0.124 |
| HEMISPHERE*PRIME | | 0.14 | 2 | 0.07 | 8.039 | 0.001 |
| HEMISPHERE*PRIME* meta-d' | | 0.013 | 2 | 0.007 | 0.754 | 0.478 |
